# Cannabinoid Hyperemesis Syndrome - A Case Report of an uncommon condition

**DOI:** 10.1192/j.eurpsy.2023.1615

**Published:** 2023-07-19

**Authors:** B. Peixoto, M. Bicho, M. Cruz, M. Mendonça, V. Ustares

**Affiliations:** Psychiatry, Hospital do Divino Espírito Santo, Ponta Delgada, Portugal

## Abstract

**Introduction:**

The increasing prevalence of cannabis use in the world requires awareness of cannabis-related disorders such as cannabinoid hyperemesis syndrome (CHS). CHS is characterized by cyclic episodes of nausea, vomiting and abdominal pain, affecting chronic cannabis users, who usually recur to hot showers to relief the symptoms. The pathophysiology underlying this syndrome is still unclear. Despite the well-established anti-emetic properties of cannabis, there is increasing evidence of its paradoxical effects on the gastrointestinal tract and central nervous system.

**Objectives:**

The authors pretend to inform the readers about the rare Cannabinoid Hyperemesis Syndrome (CHS).

**Methods:**

The authors describe a case of a 22 years old patient with chronic cannabis use, cyclic and intractable nausea and vomiting, to bring to the attention that this condition exists and is underdiagnosed.

**Results:**

CHS should be strongly considered in the differential diagnosis of persistent vomiting in patients who reports relief with hot showers. In the acute setting, supportive care with intravenous fluids, dopamine antagonists, topical capsaicin cream, and avoidance of narcotic medications has shown some benefit. However, cannabis cessation appears to be the best treatment.

**Conclusions:**

CHS prevalence will continue to rise in parallel with increasing worldwide cannabis use and potency. So, health professionals must be aware of this syndrome, its diagnosis, and treatment, to provide better care and avoid overlooking CHS. Further research is required to elicit the exact mechanism and additional therapies for this condition.

**Disclosure of Interest:**

None Declared

